# Healthcare provider and patient perspectives on access to and management of atrial fibrillation in the Northern Province, Sri Lanka: a rapid evaluation of barriers and facilitators to care

**DOI:** 10.1186/s12913-022-08440-1

**Published:** 2022-08-23

**Authors:** Vethanayagam Antony Sheron, Shivany Shanmugathas, Tiffany E. Gooden, Mahesan Guruparan, Balachandran Kumarendran, Gregory Y. H. Lip, Semira Manaseki-Holland, Krishnarajah Nirantharakumar, Kaneshamoorthy Shribavan, Kumaran Subaschandren, Rashan Haniffa, Rajendra Surenthirakumaran, G. Neil Thomas, Powsiga Uruthirakumar, Sheila Greenfield, Deirdre A. Lane, Abi Beane, NIHR Global Health Research Group on Atrial Fibrillation Management, Vethanayagam Antony Sheron, Shivany Shanmugathas, Tiffany E. Gooden, Mahesan Guruparan, Balachandran Kumarendran, Gregory Y. H. Lip, Semira Manaseki-Holland, Krishnarajah Nirantharakumar, Kaneshamoorthy Shribavan, Kumaran Subaschandren, Rashan Haniffa, Rajendra Surenthirakumaran, G. Neil Thomas, Powsiga Uruthirakumar, Sheila Greenfield, Deirdre A. Lane, Abi Beane, Ajini Arasalingam, Isabela M. Bensenor, Peter Brocklehurst, Kar Keung Cheng, Wahbi El-Bouri, Mei Feng, Alessandra C. Goulart, Yutao Guo, Gustavo Gusso, Lindsey Humphreys, Kate Jolly, Sue Jowett, Chamira Kodippily, Emma Lancashire, Xuewen Li, Yan-guang Li, Trudie Lobban, Paulo A. Lotufo, David Moore, Rodrigo D. Olmos, Elisabete Paschoal, Paskaran Pirasanth, Uruthirakumar Powsiga, Carla Romagnolli, Itamar S. Santos, Alena Shantsila, Isabelle Szmigin, Meihui Tai, Timo Toippa, Ana C. Varella, Hao Wang, Jingya Wang, Hui Zhang, Jiaoyue Zhong

**Affiliations:** 1 Department of Community and Family Medicine, Faculty of Medicine, University of Jaffna, Jaffna, Sri Lanka; 2 Department of Marketing, Faculty of Management Studies and Commerce, University of Jaffna, Jaffna, Sri Lanka; 3 Institute of Applied Health Research, University of Birmingham, Birmingham, UK; 4 Department of Cardiology, Teaching Hospital, Jaffna, Sri Lanka; 5 Liverpool Centre for Cardiovascular Science, University of Liverpool and Liverpool Heart and Chest Hospital, Liverpool, UK; 6 Centre for Inflammation Research, University of Edinburgh, Edinburgh, UK

**Keywords:** Atrial fibrillation, Care pathways, Chronic disease management, Barriers to care, Decision making, Health seeking behaviours, Qualitative methods, Rapid evaluation

## Abstract

**Background:**

Atrial fibrillation (AF) is the most common cardiac arrhythmia that affects 60 million people worldwide. Limited evidence on AF management exists from low- and middle-income countries and none from Sri Lanka. We aimed to investigate the existing AF care pathway and patients’ perception on AF management to identify barriers and enablers for optimal AF care in Northern Province, Sri Lanka.

**Methods:**

A rapid evaluation was undertaken with use of qualitative methods. Local healthcare providers (HCPs) mapped the intended pathway of care for AF patients which was then explored and annotated through 12 iterative sessions with additional HCPs. Topics of inefficiencies identified from the finalised map were used to guide focus group discussions (FGDs) with AF patients. AF patients who were attending the anticoagulation clinic at the only tertiary hospital in Northern Province were recruited and invited to participate using purposive sampling. The topic guide was developed in collaboration with local clinicians and qualitative experts. FGDs were conducted in the native Tamil language and all sessions were recorded, transcribed verbatim and thematically analysed using a deductive approach.

**Results:**

The mapped pathway revealed inefficiencies in referral, diagnosis and ongoing management. These were explored through three FGDs conducted with 25 AF patients aged 25 to 70 years. Two key themes that contributed to and resulted in delays in accessing care and ongoing management were health seeking behaviours and atomistic healthcare structures. Four cross-cutting sub-themes identified were decision making, paternalistic approach to care, cost impacts and lifestyle impacts. These are discussed across 10 unique categories with consideration of the local context.

**Conclusions:**

Strengthening primary healthcare services, improving public health literacy regarding AF and building patient autonomy whilst understanding the importance of their daily life and family involvement may be advantageous in tackling the inefficiencies in the current AF care pathway in Sri Lanka.

**Supplementary Information:**

The online version contains supplementary material available at 10.1186/s12913-022-08440-1.

## Introduction

Atrial fibrillation (AF) is a common risk factor for stroke and increases economic burden due to associated morbidity and mortality [1]. It is estimated that case prevalence of AF has increased two-fold in the last 30 years and is now estimated at 60 million globally [2]. AF increases the risk of stroke five-fold and also increases the risk of heart failure, hospitalisation and death [3]. High-income countries have the highest reported prevalence of AF (2–4% in the adult population) [4]; however, AF prevalence is likely underestimated in low- and middle-income countries (LMICs), in part due to, limited knowledge and awareness of AF and access to diagnostics [5]. The “ABC pathway of care” for AF is well established in many high-income countries and could potentially enhance management in LMICs [6]. The ABC pathway proposes to avoid stroke by anticoagulation (A), achieve better symptom management by the use of rate and rhythm control strategies (B), and optimise management of cardiovascular conditions or other comorbidities through medical therapy and lifestyle changes (C) [7]. Stroke prevention with warfarin, which is widely available in many under resourced health systems, is evidenced to reduce the risk of stroke by up to 67% [8]. In addition, in many developing healthcare systems, there is increasing awareness of non-communicable disease management, and growing investment in secondary care services, thereby increasing access to and availability of specialist medical, cardiology care and diagnostic services, including imaging. However there remains limited connectivity of care pathways between primary and secondary services [9], which are necessary for successful long-term management of complex concomitant diseases to which AF contributes. Access to, and uptake of, services varies widely internationally, and contributes to ongoing inequalities in universal access and in quality of care for patients [10].

Sri Lanka, a South Asian LMIC with a population of approximately 22 million [11], has a well-established government-led national health service. Unprecedented is the country's track record in the management of communicable diseases and in exceeding its South Asian neighbours in maternal and child health development goals [12]. Life expectancy is increasing and achieving these health metrics has been in part due to well organised public health programmes. However, over the last 5 years community health needs have begun to change. Like many countries in the region, non-communicable diseases and specifically cardiovascular diseases (e.g. AF, stroke, hypertension and ischaemic heart disease) are rising [13]. The prevalence of AF is currently unknown in Sri Lanka; although, the burden of stroke is high, and current strategies such as the ABC pathway are not commonly employed [14, 15]. Within neighbouring China, strategies including the ABC framework and raising the profile and understanding of the disease within the healthcare professional (HCP) and public communities have proven effective at improving timely recognition and management of AF [16]. We formed a collaboration of cardiology specialists, primary care physicians and researchers to seek a better understanding of patients’ and HCPs’ perspectives of the current pathways of care for AF in Northern Province Sri Lanka, and to identify facilitators and barriers to early diagnosis and ongoing management and to identify opportunities to improve quality of care provision.

## Methods

### Study design

Rapid evaluation (RE) methods [17] were used to explore and understand the existing care pathways [18] in Northern Province, Sri Lanka. RE methods are increasingly used in healthcare improvement because they provide a pragmatic and stakeholder-centric approach to exploring the complexities of service provision, social and cultural factors shaping healthcare delivery, and nuanced practices of care provision in short time frames [17]. Attaining understanding of the context in which care is delivered allows tailoring and embedding of any subsequent service improvement interventions with increased opportunity for their success. RE is characterised by intensive, team-based investigation that uses multiple methods of data collection; has an iterative process for collection and analysis; and follows the principles of participatory action to quickly develop a holistic understanding of a program from the perspectives of key stakeholders [17]. Stakeholder-led research can provide insight into specific, complex processes and systems from locally-defined perspectives with the intention of subsequently implementing change [19]. This RE was co-designed with existing AF care providers and trained community healthcare workers. Participatory research methods were chosen to foster a shared understanding of local practice and identify barriers and enablers experienced by both HCPs and patients.

Pathway mapping provided the preliminary framework through which to understand the existing pathways of care within both community and hospital settings [20, 21]. Following the pathway mapping, focus group discussions (FGDs) were used to elicit patients’ experiences of accessing AF services, from initial diagnosis through ongoing chronic management [22]. A reflexive process, whereby complex aspects of the existing care pathways, identified in the mapping, were used to guide the group discussions regarding barriers and facilitators to care [21].

Ethical approval was received from the Ethics Review Committee of the Faculty of Medicine at the University of Jaffna (approval number J/ERC/19/107/NDR/0218). All participants provided written informed consent. Patients were not involved in the design or development of this study.

### Study setting and historical context of care provision

Northern Province, Sri Lanka includes five districts and has a population of 1.25 million [23]. Jaffna Teaching Hospital (JTH), the only tertiary hospital in the Northern Province was established in 1850 and has dedicated outpatient clinics for providing combined care for patients with cardiovascular disease including those with AF. These clinics run weekly and are attended by over 150 patients each week. Interventional services including ablation, surgery and imaging were established in 2000 along with 24-h emergency and critical care services. Prior to this, timely access to specialist care was largely absent in the province, with patients being required to travel to Colombo (approximately 220 miles south-west and a journey of 10–12 h by road or train). During the civil war in Sri Lanka (July 1983 – May 2009) the Northern Province was a central location of the conflict. Travel was further affected by restrictions, with the only access to services requiring boat journeys which were described as lasting several days, and which were both physically challenging and financially costly. These lived experiences, incredibly influential and with long-lasting impact for those patients, are fortunately not reflective of the current healthcare system and as such are reported here as context.

### Data collection

#### Pathway mapping

Pathway mapping sessions were held over a six-week period from June to August 2020. Primary, secondary and tertiary care centres in Jaffna where patients with known AF attend inpatient and outpatient care (medicine, cardiology wards, outpatient clinics for cardiology, stroke, anticoagulation) or departments where patients may present with new symptoms (emergency departments, acute admission wards), were identified by the research team. A representative of consultants, medical officers, nursing officers and healthcare assistants were purposefully selected from these clinical areas and invited to participate in the pathway mapping. Participants were first asked to describe (i.e. map) the intended pathway of care for patients accessing care for AF, both as an emergency presentation to JTH and following referral from community care providers. Maps of the pathways were initially sketched onto large sheets of paper and HCPs were then asked to map points in the pathway where patients might be asked to access different services within the healthcare system (e.g. primary care units, emergency departments, outpatient clinics, inpatient wards and diagnostic departments including echocardiography, radiology and laboratory services). This process was continued through inpatient stay, discharge and follow up care for outpatients. Once the care continuum was mapped out, additional HCPs were invited to review the initial mapping. Variations in perspectives were identified and annotated and used to refine and enrich the map of pathways as perceived by the stakeholders. This process of review, refinement and enrichment was then repeated with both hospital and community-based care teams with expertise in AF management. In total, 12 pathway mapping sessions were completed.

#### Focus group discussions

Known AF patients identified through attendance at the anticoagulation clinic were approached by members of the research team (BK, VAS, SS, PU and KS) and invited to participate in the FGDs. Patients were provided with information leaflets and given the opportunity to ask questions regarding participation, prior to giving written consent. Participants were selected using purposive sampling to ensure diversity in characteristics such as age, sex, socio-economic status and comorbidities. A topic guide was drafted first in English, refined by the research team (including clinicians based in JTH) and by qualitative research experts (SG, DAL and AB). The topic guide was informed by the commonalities and variances in access to care and ongoing management of AF identified during the pathway mapping. In addition, participants were asked to describe their understanding of the disease. The finalised FGD topic guide was translated to Tamil by a bilingual expert and retranslated to English by a different bilingual expert to check for accuracy (Supplementary File 1).

The FGDs were conducted in a private room near one of the clinics within JTH. The location was selected for its proximity, ease of access to patients and privacy. Participants were seated at a round table and joined by members of the research team (BK, VAS, SS, PU and KS). Following initial introductions and scene setting regarding the purpose of the research, the researchers initiated conversation using the topic guide. FGDs, which included a mix of participants based on sex, age and socio-economic status (defined by urban or rural living) were carried out in the local language of Tamil and audio-recorded using devices visible in the room and supplemented by contemporaneous note-taking. The latter were used to enrich the audio-recordings and provided documentation of body language and non-verbal interactions during the discussions. Recordings were transcribed in Tamil and translated into English by two independent bilingual experts. Transcripts were read alongside the notes captured by researchers (BK and SS) to check for variation and consistency and to add context to the transcripts for analysis.

### Data analysis

#### Pathway mapping

Data collection and analysis occurred iteratively and in parallel to the mapping sessions. This approach of analysis and data collection in tandem, led by multidisciplinary research teams, is indicative of RE, promoting rapid a generation of themes and encouraging a focus on action-orientated recommendations. The finalised map was then reviewed, consolidated and reproduced using digital software [24].

#### Focus group discussions

Transcripts of the FGDs were deductively analysed independently by three researchers (VAS, SS and TEG) to identify the key concepts (themes) regarding diagnosis, referral and management and any barriers and facilitators of each. VAS and SS have extensive experience of working in and around the Sri Lankan healthcare system, including in organisational and community healthcare settings in Jaffna. TEG is a research fellow and PhD candidate at the University of Birmingham trained and experienced in qualitative methods. Initially the text was open-coded by reviewing all text line by line and then descriptive codes were assigned to the words, sentences and paragraphs in the transcripts. At this stage of the analysis, lines in the transcripts were linked and grouped as the first set of codes which related to the current clinical pathway and referral practices for AF patients. Axial coding in the next step of the data reduction process linked the descriptive codes via repackaging and combined the data to identify categories that have similar characteristics [25]. After developing categories, the relationships between the categories were explored to reveal higher level themes. For example, initial analysis of the scripts revealed how social obligations and an individual's faith influence their decision making regarding accessing both initial and ongoing treatment. These “categories”, and related categories of the “role of the family” and wider “collective decision making” emerged as the subcategory “decision making” which contributed to overarching theme of “health seeking behaviours”.

### Patient and public involvement

Patients were not involved in the recruitment for or design and conduct of the study. However, the topic guides developed were piloted with three AF patients which resulted in only minor modifications to the materials. The results of this study will be disseminated directly to all participants and to other AF patients through information posters at anticoagulation and medical clinics located at JTH.

## Results

### Pathway mapping

**Fig. 1:**
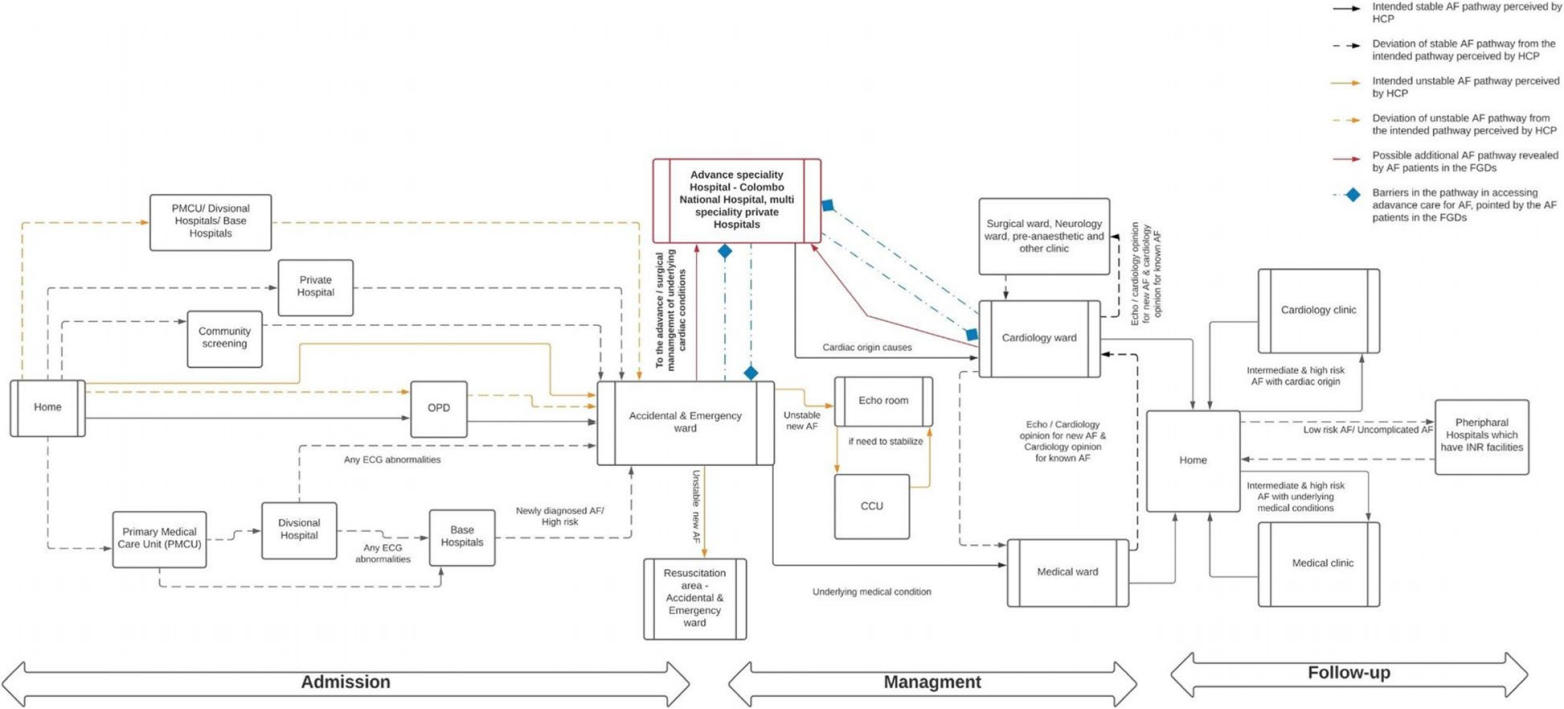
AF pathway in Sri Lanka; findings from the pathway mapping exercise by healthcare providers

Forty-three HCPs were invited to participate in the pathway mapping, and all agreed to participate. The care pathway mapped by stakeholders is described in Fig. 1. Deviations and inefficiencies throughout the pathway were revealed, but were dominant at points in the pathway where community and primary care interfaced with tertiary and specialist care, resulting in perceived delays in timely diagnosis, and again as part of ongoing management (e.g. therapeutic titration of anticoagulation medication, a mainstay of AF management in the region). Health service organisational structures and health seeking behaviours were identified as contributing drivers to these alternative care pathways and explored in further detail below.

### Focus group discussions

Twenty-five patients with a known diagnosis of AF were invited to participate in the FGDs and all agreed to participate. Three FGDs each lasting approximately 90 min were conducted with groups of 10, 7 and 8, respectively. Participant demographics, including employment, commodities and home districts are provided in Table 1. Most participants were aged 65 years and older (*n* = 11; 44%), female (*n* = 18; 72%) and married (*n* = 14; 56%). Demographics across the three FGDs were similar regarding age, sex and socio-economic status (i.e. rural/urban address).

**Table 1:** Participant characteristics from each focus group discussion

Participant number	FGD number	Age group	Sex	Marital status	Employment	Comorbidities	Socio-economic status
1	1	65 +	Male	Married	Employed	COPD	Urban
2	1	55–64	Female	Widowed	Housewife	DM	Urban
3	1	65 +	Female	Single	Unemployed	IHD, VHD	Rural
4	1	45–54	Female	Married	Housewife	RHD	Urban
5	1	< 45	Female	Single	Unemployed	–	Rural
6	1	55–64	Male	Married	Retired	IHD, RHD	Rural
7	1	55–64	Female	Married	Housewife	VHD	Rural
8	1	< 45	Female	Married	Employed	VHD, COPD	Rural
9	1	55–64	Female	Widowed	Housewife	VHD, HT	Urban
10	1	65 +	Male	Widowed	Retired	COPD	Rural
11	2	65 +	Male	Married	Retired	VHD, CHF	Rural
12	2	65 +	Male	Widowed	Employed	–	Urban
13	2	65 +	Female	Widowed	Housewife	DM	Urban
14	2	55–64	Female	Married	Housewife	VHD	Rural
15	2	45–54	Female	Married	Employed	VHD, DM	Urban
16	2	65 +	Female	Widowed	Housewife	HT, DM	Urban
17	2	< 45	Female	Married	Housewife	VHD	Rural
18	3	65 +	Female	Married	Housewife	IHD, RHD	Rural
19	3	65 +	Female	Married	Housewife	VHD, HT, BA	Urban
20	3	< 45	Male	Widowed	Employed	RHD, DM	Urban
21	3	65 +	Male	Married	Retired	VHD, HT, BA	Rural
22	3	55–64	Female	Married	Housewife	HT, COPD	Urban
23	3	45–54	Female	Widowed	Housewife	IHD, RHD	Rural
24	3	< 45	Female	Married	Employed	DM	Rural
25	3	65 +	Female	Widowed	Housewife	IHD, RHD, HT	Urban

*BA* Bronchial asthma, *CHF* Congestive heart failure, *COPD* Chronic obstructive pulmonary disease, *DM* Diabetes mellitus, *FGD* Focus group discussion, *HT* Hypertension, *IHD* Ischaemic heart disease, *RHD* Rheumatic heart disease, *VHD* Valvular heart disease

**Fig. 2:**
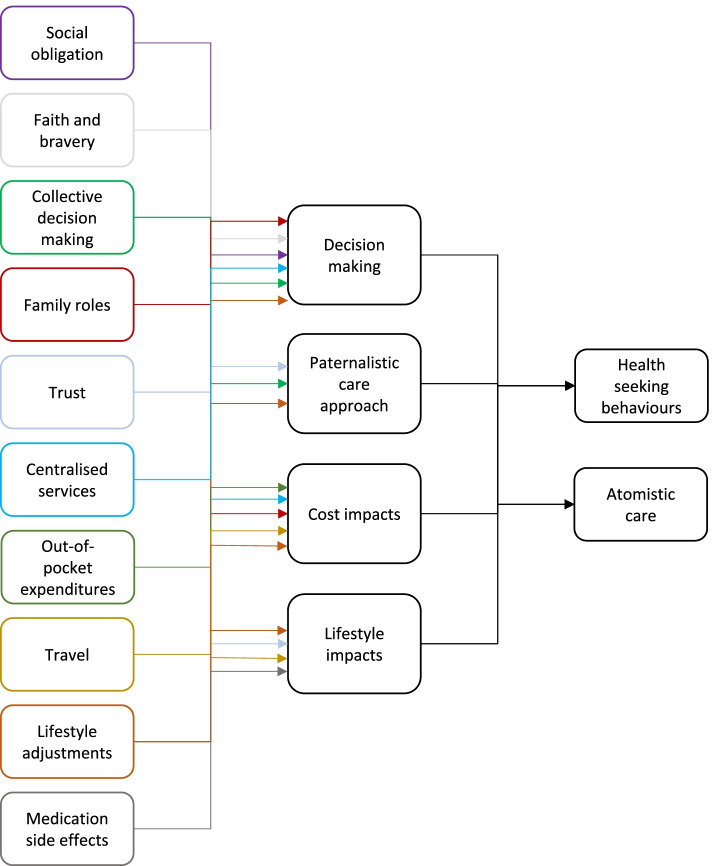
Coding categories and their interconnected themes and sub-themes

Initial open coding of the transcripts revealed 33 descriptive sub-categories, which were subsequently repackaged to form 10 categories. Categories were then linked with common themes and to the key findings of the patient pathway. Two overarching themes were identified that impeded and facilitated initial access to AF diagnosis, ongoing management, or both: (i) health seeking behaviours and (ii) atomistic healthcare structures. Both themes are underpinned by four cross-cutting sub-themes: (i) decision making, (ii) paternalistic approach to care, (iii) cost impacts and (iv) lifestyle impacts (see Fig. 2).

#### Decision making sub-theme

*Decision making* describes the complex relationships between patients, families and peers and explores social constructs such as *social obligation, family roles* and *collective decision making*. Cost and access to care (related to *travel*) were also mentioned as influences on decision making regarding both presentation and ongoing management.

Participants were asked to reflect on what they were experiencing at the time of AF onset. Symptoms described combined both acute presentation and ongoing, even escalating symptoms, such as tiredness, breathing difficulties, increased palpitations, excessive weight loss, frequent fever and giddiness. Despite these escalating, severe clinical symptoms, several participants described reluctance to access healthcare until these symptoms impacted on their ability to work and their wider activities of daily living.

*“I did not feel good during the night … it happened between four to five days, however, I consulted with the doctor on the 4.*^*th*^*-time attack.” (FGD 1, P2)*

One participant described how these broader symptoms had been misdiagnosed at initial presentation as an endemic and seasonal condition.

*“I had a fever… at hospital they gave Panadol only, they thought it was dengue, but the result was negative after test…” (FGD 1, P3)*

Participants reported that they were more likely to decide to seek healthcare if their symptoms were directly cardiac in nature compared to those who experienced more generic symptoms, including shortness of breath and or flushing/fever.

*“Had fainting attacks, 2002 when took to [district hospital], transferred to [JTH] because of hole in the heart and [consultant cardiologist] did [surgery].” (FGD 2, P4)*

*“I used to carry water pot, one day while I was carrying water pot I felt too ill and shortness of breath, after [death of husband] I became like this due to the worries, then this leg had small swelling, in that time only I came to [hospital] with shortness of breath.” (FGD 3, P1).*

It was often family members or consultation with trusted peers that prompted healthcare being sought. Across all FGDs, it was family members (often younger members of the family) or close friends who encouraged the person to go to hospital and seek care *(family roles*).

*“I felt difficulties in respiration for some days. My younger brother’s wife who was a doctor told me to consult the consultant, She used to say this for long.” (FGD 1, P3)*

*“I felt tired after sweating, I sat in a place where I felt invisible, I didn't have any disease, I told my younger sister. She asked me “why are you having this much of sweat and I am not having” and asked me to go to the hospital.” (FGD 1, P9).*

Whilst there was no explicit expectation on participants to discuss health-related decisions with their peers or family, decision making to access care, and ongoing management of AF (*collective decision making)* was clearly favoured over individual decisions and in many instances, guidance and reassurance from family/peers was a facilitator to accessing health services.

*“I told my daughter after the disease came, she worked here at hospital as a midwife, I told her “I can’t do anything”. She told “pay the money in [hospital], the doctor will come on Saturday, he will take you to the ward.” As I did what she told me, I am alive.” (FGD 2, P2).*

*“I asked, “Are there any problems due to the operation?” And I told them after discussing with family members only I can tell.” (FGD 1, P9)*

Whilst social factors (e.g., strong, supportive, extended family connections) had a positive impact on many patients’ health seeking behaviour, for some, societal factors were a potential barrier. Some participants risked missing or delaying uptake in treatment due to social obligations, such as attending family events and funerals. In such instances, it was the positive influence of the relationship with, and trust in, clinicians that mitigated these delays (*paternalistic care and trust*). These categories are explored further below in the sub-theme of paternalistic approach to care.

*“He told to come to ward on tomorrow, I said my cousin passed away, I will finish the rituals and come back on Monday, but he said strictly you have to come, that’s why I have been alive for eight years … it was good as I didn’t attend the funeral … I accepted his words and went to the ward … If I disagreed his word my condition might be worsened.” (FGD 2, P2).*

#### Paternalistic approach to care sub-theme

*Paternalistic approach to care* refers to the relationships between patients and HCPs (specifically the role and position doctors have in the community) and the relationship and *trust* between doctor and patient. This theme also encapsulates *faith and bravery*, a category which describes a strong sense of higher or external power in which participants trust that they will be well.

Throughout all FGDs, participants' description of seeking care and their ongoing health seeking behaviour was heavily influenced by their relationship with the doctor. Participants described a paternalistic approach to care, where doctors' decision making was divisive in their health seeking behaviour and treatment choices. These relationships often manifested with signs of absolute trust in the doctor's instructions, and a huge sense of gratitude for the care received, both at the time of initial AF management and longer-term management.

*“I don’t eat anything suggested to avoid by the doctors, otherwise no food restriction, they asked me to walk, I used to walk for two years following the operation…” (FG1 P7)*

*“I don’t use [warfarin], I have to inform the doctors, after informing [the doctor] only we can do anything…” (FG1 P6)*

*“If I have any problem I see [consultant], he tells the needed, we adopt as he tells.” (FGD 2, P8)*

#### Cost impacts sub-theme

*Cost impacts* describe not only the out-of-pocket expenditure and loss of income experienced by participants but also the categories of “*travel*” and “*centralised services*” as these cost the patients an abundance of time and effort to receive care. Together these factors were a barrier to seeking healthcare and attending hospital for ongoing management.

*“Madam [doctor] told “… there are no facilities for the surgery, thus you have to go to Colombo” needed money for that and transport limitation also there. Furthermore, that was wartime, in that situation I didn’t consider this as serious.” (FGD 1, P8).*

*“They gave warfarin for two to three years, then they asked, “Do you like to do operation, if you do operation only you can live longer with your children.” I replied I don’t have much money.” (FGD 2, P7).*

This sub-theme explores the lived experience of the pluralistic care pathways that exist in the province, and how for some patients this resulted in increased cost, possible misdiagnosis and delays in treatment. Participants described how they accessed several HCPs, including private healthcare professionals, either before or in parallel to accessing government led cardiac care services.

*“I had fever, visited at [divisional hospital] … they thought it was dengue, but the result was negative … Prior to the Fever I had tiredness, if I work, I feel tired, difficult in respiration … [divisional hospital] thought this was wheezing and send to [JTH], after testing they said “there is a 40% block”.” (FGD 1, P3).*

*“Went to a private hospital they told, “you have problem in heartbeat. I will give a letter, consult at Jaffna Teaching Hospital.” They transferred me to [JTH] and diagnosed a valvular problem. For the valvular problem they asked to do the operation at Colombo.” (FGD 2, P5).*

In several instances these *centralised services* resulted in patients incurring lengthy travel and financial costs and delays in specialist assessment.

*“3 to 6 times went to Colombo, they neglected to do, then we informed our children to get money. [the children] got money and did it privately.” (FGD 2, P1)*

Once the decision to access services was made, participants described experiencing long waiting times and the need for repeated visits to the hospital and consequently increased travel costs and a loss of working time for patients and family members, who often accompanied their relative to appointments.

*“Here the waiting time is high … we have to wait until 1 pm and then only we can go home.” (FGD 1, P8)*

*“We are coming [to JTH] because INR [international normalised ratio] can’t be checked in other places… If INR facilities is [at divisional hospital] and if a doctor will see us and we are able to visit there we can save the expenditure for the transport and time too.” (FGD 3, P3).*

#### Lifestyle impacts sub-theme

Participants in all FGDs described significant adjustment and disruption to their day-to-day activities resulting from their AF management. These lifestyle adjustments were predominantly restrictive in nature, permeating diet, daily activities, and limiting family and grandchildren interactions and resulted in limitations in travel and attending important social engagements (*life impacts*).

*“Should not carry small child even baby also … Should not cut by knife, should not cut vegetables.” (FGD 2, P1)*

*“They asked to stop riding motorbikes, because if any wound appears it won’t heal, and not to do any work at home.” (FGD 1, P10)*

*“Used to go to funerals, might be due to that vibrations, now I don’t go inside.” (FGD 2, P4)*

*“Can’t go anywhere, I have to avoid it. Even if it is important … Should not hear loud sounds/vibration, it will worsen the condition.” (FGD 3, P8)*

Whilst the paternalistic approach to care and the relationship of *trust* between patient and doctor was viewed positively by patients, there was some evidence that participants felt unable to challenge doctors’ instructions even when such instructions felt unrealistic or would result in limiting their social interactions and impacting their wellbeing. Unwillingness to challenge doctors’ decision making, was sometimes more influential than the potential consequences of treatment, and the actual consequences of financial burden *(cost impacts)* for both the patient and their family.

*“….We avoid leafy vegetables as they informed, if we want to cure our disease we have to obey what they are saying…” (FGD 3, P5)*

*“I had two options, one I will die here without doing anything. Second I can survive if I agree for the surgery without considering the risk.” (FGD 1, P8)*

*“They asked me to go to Colombo soon, I refused as have to spend money and requested [the doctor] to send me [to a different] hospital, he replied “I will take nearly one year to do the operation. If we send, do the operation without considering the money.” Then my son booked in [private hospital] in Jaffna.” (FGD 2, P6).*

In addition to trust in the doctors' guidance and the positive influence family members had in guiding patients when seeking care, discussions revealed that *faith and bravery* also influenced care. This self-perception of bravery positively influenced both patients’ outlook on their diagnosis and adherence to therapy.

*“I am brave, everyone said don’t do as this is a serious operation, but I went…” (FGD 3, P5)*

*“Everyone knows, everyone is telling “Thunintha Kaddai” [Tamil phrase for ‘I am brave’].” (FGD 1, P4)*

Faith was, at least for some participants, an equally important influence on care as medical advice, and influenced participants’ perception of the success of treatment and offset the potential barriers, of increased travel and financial costs.

*“Half of the treatment is faith, whereas treatment is only the other half. If we see the right thing as right only it will appear as right. If you understand correctly what to eat and what not to eat then it will be okay.” (FGD 1, P8).*

*“… went to Colombo for two times to do the surgery because of faith.” (FGD 1, P8)*

## Discussion

This study reports expected and intended pathways of care as described by HCPs and describes patients' perceptions of the current barriers and facilitators to referral, diagnosis, and ongoing management for AF care. Two interrelated themes, *‘atomistic care pathways’ and ‘health seeking behaviours’* together with their sub-themes of *decision making, paternalistic care approach, cost impacts* and *lifestyle impacts* were revealed and together these encapsulate both barriers and facilitators to initial diagnosis and ongoing management of AF in Northern Province, Sri Lanka. The impact of these system based and socio-cultural factors on healthcare efficiency and quality of life for patients is discussed further, along with recommendations for future healthcare improvements in the region.

### Atomistic care pathways

The last decade has seen significant investment in specialty cardiology care, including diagnostics, imaging and interventional services in Sri Lanka [12]. Whilst investment in these centralised services has been essential for the improved provision of acute cardiovascular disease care in the region, the underlying health system infrastructure needed for providing continuity of care across primary and tertiary care remains underdeveloped. Differences between intended and actual care pathways experienced by HCPs and patients result in inefficiencies in care, increased costs and lifestyle impacts; affecting both patients for whom AF is a symptom of underlying valvular disease and increasingly prevalent in Asia and patients presenting with AF as a consequence of risking non-communicable diseases. Despite the intended route of referral and assessment being via primary care, to direct referral to outpatient cardiology services, stakeholders described how patients accessed care through multiple providers (traditional and modern) and multiple systems (private and public) (Fig. 1).

Like most healthcare systems in Asia, Sri Lanka’s is increasingly pluralistic, having both private and public services available and patients increasingly choosing to navigate both, often contemporaneously [26]. As reported in other studies, participants in the present study associated private care with increased convenience, increased efficiency and greater quality of care compared to government facilities [27]. Whilst choice of care providers is arguably important for patients, and for enabling governments to achieve universal health coverage, the duality of the services can, without investment and governance, risk hampering timeliness of accessing acute care services and potentiate poor decision making [26]. Private healthcare systems in LMICs are often fragmented and with limited infrastructure to assess service delivery quality and their rapid emergence has not been matched with public health literacy to navigate such systems [26]. Despite the diversity and complexity of referral care pathways for AF, most patients in Northern Provence, Sri Lanka described attending centralised hospital-based services for their ongoing disease management. For many patients (and their families), the location and organisation of hospital care provision resulted in considerable burden due to travel, rising out-of-pocket expenditure, long waiting times and disruption to their and their families’ daily lives. Whilst centralisation of services is both necessary and potentially desirable for health service improvement and sustainability, such system shifts can further disenfranchise vulnerable sectors of the community, and may in the context of AF, have unintended consequences (cost, inefficiency), deleterious to care quality [27, 28].

### Health seeking behaviours

This qualitative study offers unique insight into patients' perspectives and first-hand experiences regarding management of AF care in Sri Lanka. The factors influencing individuals’ motivations to seek AF care reported in this study are encapsulated by the Health Belief Model [29], which proposes that the perception of a personal health behaviour threat is influenced by at least three factors: *individual perceptions of health, wellbeing, disease severity and symptoms; modifying factors (including demographics and social cues)*; and *likelihood of action (perceived benefit of seeking treatment in relation to perceived threats)* [29, 30].

Increasing severity of symptoms prompted patients to seek investigation and treatment for AF. Misinterpretation of AF symptoms, mistaken for better known communicable diseases endemic in the region (e.g. dengue), resulted in misdiagnosis and hesitation from patients in seeking treatment. Whilst vertical public health education programmes in Sri Lanka have been hugely successful in managing communicable diseases such as dengue, patient and public awareness of non-communicable diseases is poorer [31]. Lack of awareness regarding AF and its associated risks is a known impediment to initiating AF care and may mislead patients into perceiving barriers (i.e. costs, travel) to outweigh the potential benefits from seeking care [32].

Descriptions of the importance of family members (often children or younger generation) being involved in decision making was often the cue to action for patients (parents and grandparents) seeking care. Patients' children placed greater importance on health and wellbeing than patients themselves, who instead expressed concerns over social obligations and economic barriers. Increasingly acknowledged in health seeking behaviours is the role family members play in instigating referral (lay-referral) and sustaining ongoing engagement in treatment [33]. As such, family and community members have an important role in facilitating and overcoming barriers to care.

HCPs were also important in actioning referral and ongoing AF management. Patients described overwhelmingly the positive perceptions of *trust* for their HCPs guidance in decision making, even if adherence to treatments negatively impacted the patient’s quality of life. Paternalistic care structures are often observed in the context of care for older patients or when health literacy is limited [34–36]. Whilst such approaches to care may at first appear benevolent, and as perceived here, appear to facilitate concordance with therapy, such approaches are also unlikely to promote patients’ self- efficacy. Self- efficacy, whereby patients have self confidence in their knowledge and management of their wellbeing is increasingly considered important in improving quality of non-communicable disease care [37]. Increasing patients’ self-efficacy (or agency) is associated with patients being more likely to perceive their disease more seriously, recognise declining physical health, and increase the likelihood of implementing lifestyle changes to prevent or limit disease progression [37, 38].

### Strengths and limitations

The main strength of our study was the inclusion of both HCPs and patient views on the pathway of care for AF. Bringing together perspectives of diverse stakeholders has enriched our findings and ensured that the barriers and potential opportunities for improving the health system processes are reflective of the priorities for both patients and care providers. The combination of health system research methods used here was another strength: pathway mapping enabled us to develop a comprehensive understanding of the current pathways in which AF patients receive a diagnosis and receive on-going management. The FGDs complemented these findings by enriching our understanding of stakeholders’ perceptions as to the drivers to barriers in care. This multimodal research was led by clinician- researchers with an in-depth understanding of the contexts within the health system and partnered with international research colleagues which encouraged reflexivity during the analysis. This study could have been further strengthened by including stakeholders representing administrative and other applied health professionals. We intend to take the recommendations of this research forward to this wider stakeholder group, prior to the development of potential interventions for improvement. Furthermore, the study was conducted in only one region of Sri Lanka (i.e. Northern Provence) and therefore the findings may not be generalisable to other regions of Sri Lanka.

### Recommendations

Changing epidemiology and burden of non-communicable disease in Sri Lanka (as is more widely reported in South Asia), requires not only a reconfiguration of healthcare delivery but greater investment in primary care services, and in public knowledge and awareness from multiple stakeholder providers. Primary care services, when well-resourced and well-equipped have proven central to effective management of non-communicable diseases [39]. Shifting the focus of AF management away from the hospital setting to the community could relieve the burden on the already overstretched acute care sector, and avoid many of the *lifestyle impacts* (travel, out of pocket expenditure, social obligation) described here by patients [40]. Furthermore, successful longer-term management of non-communicable diseases, will likely require private and public HCPs to work together in multiple stakeholder partnerships to seek both new and novel curative therapies and to raise the profile of non-communicable diseases. National science and research funding bodies are well placed to help lead such initiatives, generating not only new evidence, but also increasing public awareness of the importance of such diseases and their impact on social and economic growth.

Drawing on the Health Belief Model [29] we understand how responses to illness and adherence to lifestyle changes are influenced by patients’ perceptions of the severity of the illness and the perceived consequences of not engaging with healthcare treatment, and internal (e.g. pain) and external (family, HCP, lay-referral) cues to action. In other non-communicable disease pathways including respiratory care, long-term management strategies such as telephone reminders, and community-led support groups, have been used to create “cues” serving to prompt and encourage patients and their peers into positive action [41]. Building this agency requires a shift to a shared care model, whereby patients and families are equal stakeholders in decision making. Primary care providers are well placed to lead such programmes, building on the already existing provider patient relationships.

## Conclusion

Investments in specialist services have been integral in improving access to healthcare in Northern Province of Sri Lanka. Investment in primary healthcare services is now needed to strengthen the continuity of AF care and management and may be advantageous in reducing the burden on existing acute care facilities. Greater public and community involvement is needed to increase health literacy regarding AF disease and build agency for patients living with chronic disease, to improve self-management and quality of life and enable greater decision making regarding accessing healthcare. Learning directly from the descriptions of patients’ experiences and the factors that influenced their health seeking behaviours described here could be useful in informing future primary health improvement strategies in the region.

## Supplementary Information

**Additional file 1. **Topic guide for patient focus groups about AF and anticoagulation.

## Data Availability

The datasets generated and/or analysed during the current study are not publicly available due to restrictions that apply to the availability of the data, but data are available from the corresponding author on reasonable request.

## Abbreviations

AF: Atrial fibrillation
LMICs: Low- and middle-income countries
ABC: AF Better Care
HCP: Healthcare professional
RE: Rapid evaluation
FGD: Focus group discussion
JTH: Jaffna Teaching Hospital
INR: International normalised ratio
