# Supplementary material for: Healthcare provider and patient perspectives on access to and management of atrial fibrillation in the Northern Province, Sri Lanka: a rapid evaluation of barriers and facilitators to care
Source: BMC Health Serv Res. 2022 Aug 23;22:1078. doi: 10.1186/s12913-022-08440-1 (PMC9400248; doi:10.1186/s12913-022-08440-1)
Supplement: Supplementary file 1 — Additional file 1. Topic guide for patient focus groups about AF and anticoagulation. [file 12913_2022_8440_MOESM1_ESM.pdf]

Supplementary File 1: Topic guide for patient focus groups about AF and anticoagulation

After turning on the voice recorders the facilitator starts with an introduction / warm up along the lines of:

'We have invited you here today as you have the heart condition Atrial Fibrillation and we would like to know your views on some aspects of this condition. My name is 'name' and I am here to facilitate our discussions today'

It would then be important to ask participants to briefly introduce themselves using the name they would like to be used for them during the discussions (these should already have been written on name cards / badges). Something along the lines of:

'Before we start it would be really helpful if everyone could introduce themselves to the group using the name that you would like to be used during our discussions today.'

(Indicate someone to start the process and then go round each member of the group.)

Thank the group for their introduction, set the ground rules (see ground rules for focus group set up document) and start with the first question.

Understanding and perceptions of AF

ESSENTIAL QUESTIONS

1. Can someone please tell me a bit about how you found out that you had this heart rhythm condition. (Try to encourage all group members to tell you their story- possible prompt questions might be, did that happen to anybody else, does anybody else have a different experience?)

2. What did the doctor/nurse tell you about this condition?
(Let patients have free discussion first then, if needed, use prompts to elicit understanding of different issues, specifically did they mention:
a) m*echanism* – e.g., heart doesn't work properly, heart beats irregularly/fast
b) *treatment*, i.e., it being curable/treatable

3. When you were told you had AF, how did you feel?

QUESTIONS TO INCLUDE IF TIME PERMITS / OF PARTICULAR INTEREST IN YOUR COUNTRY

4. Was any one given information about the condition to take away by the doctor/nurse?
(Prompt for details if initial response is just 'yes')

5. Did anyone look for information themselves?
(e.g., internet, books, leaflets, talking to other people, other healthcare practitioner /traditional healthcare practitioner, website address)

6. What do you think causes AF?
(e.g., other medical conditions, lifestyle behaviours (smoking, alcohol, drugs, obesity))

7. Can someone tell me what the symptoms of AF are?

(e.g., tiredness and being less able to exercise; breathlessness; feeling faint or lightheaded, chest pain; some people will not have any symptoms (this is normal) so it is important to state this if some of the participants didn't have symptoms)

8. Did you tell your family/friends/work colleagues?
(Depending on answers probe why/why not?)

Management of AF

'We have explored how you found out you had AF together with your understanding and views of the condition. Before we move on is there anything anyone would like to add? I would now like to ask some questions about how AF is managed. To start

ESSENTIAL QUESTIONS

9. Can you tell me about the different treatments you have had for AF?

(If they talk about anticoagulation and the following is not discussed prompt them about it: what they take (VKA or NOAC); why they take it; what the benefits are (they should talk about reducing stroke risk here - (probe for this if not); any concerns they have about taking anticoagulation). Similar prompts need to be used in relation to other treatments raised to explore their understanding of why they take antiarrhythmic drugs, why they had catheter ablation or cardioversion etc.; if they are not receiving any OAC, why? (personal choice, etc.)

10. Doctors prescribe anticoagulation (medicine to reduce the risk of blood clots) to manage atrial fibrillation - can anyone tell me what this is and how that it works.
(After the first response try to get other people's understanding, if no answers offer a brief correct explanation, i.e., thins blood and ask for other people's views – if incorrect answers are provided it is also important to provide an explanation to ensure groups members leave with a correct understanding. If participants say they were on it and stopped probe for the reason, i.e., own/doctors decision, side effects, bleeding, etc.).

11. Apart from any treatment you have had, do you do anything else to try to manage your condition?
(Possible probes could be: lose weight, alter diet, stop smoking/ reduce alcohol intake, exercise, control blood pressure, complementary/alternative medicine, vitamins/supplements)

12. Do you think that atrial fibrillation can put people at risk of developing other medical problems?
(Possible probe, e.g., stroke, heart failure)

Facilitator needs to say at this point: 'For the next few questions if you are not on anticoagulation treatment please think about how you might feel if you were.'

13. How did/would you feel when/if your doctor prescribed anticoagulation medication for you?

14. Are you able to tell me what medication you are on and are you happy to take the medication?
(Prompt for side effects experienced if these are not brought up naturally such as 'does anyone experience any side effects as a result of the anticoagulation medication they are taking? Or have they experienced side effects that lead to them discontinuing the anticoagulant?')

Please note that if patients mention specific drugs in response to Question 9 the facilitator could say to relevant members of the group 'You told me earlier that you take Drug X and Drug Y, do you take any other medication?....are you happy taking these medications'.....if they say yes, ask why, if they say no, ask which ones and explore why'

15. How do you remember to take your anticoagulant medication? Do you ever forget?

16. Do you think it matters whether you take the medication every day or not?

17. What do you think might happen if you took more medication or less medication than you have been prescribed?

18. How does AF affect your everyday life? (If participants talk about being symptomatic probe about how these symptoms affect their quality of life)

19. How does anticoagulation therapy affect your everyday life?

(NB It is probably important to ask separately about the two previous questions to try and elicit whether it is the condition/medication or both that have the biggest impact)

Probe: quality of life, no effects at all, have symptoms regularly, stop you doing things, have to take care, have to modify diet/alcohol, have to go to doctor more often for check-up, gives you side effects feel anxious/just carry on as normal /make you feel reassured about stroke risk.

QUESTIONS TO INCLUDE IF TIME PERMITS / OF PARTICULAR INTEREST IN YOUR COUNTRY

20. How long have you been told you should continue taking your anticoagulant?
(i.e., weeks, months or years)

Care pathways

'We talked a bit at the start about how you found out you had AF I'd like to explore that a bit more now'

ESSENTIAL QUESTIONS

21. Please explain to me who the health professionals were that you saw when you were first diagnosed with your heart condition....please can you tell me your individual stories from diagnosis to the present day.

If no one answers this you could give a possible example (e.g., I saw my GP who then wrote a letter for me to go to the hospital heart specialist....he sent me to do x and y test, then I went back to a different specialist....got z medication and was told .....

QUESTIONS TO INCLUDE IF TIME PERMITS / OF PARTICULAR INTEREST IN YOUR COUNTRY

22. How often do you have appointments for your atrial fibrillation and what kind of healthcare professionals and visits to clinics or hospitals you have to make

If no one answers this you could give a possible example e.g. I only go to the x clinic every month or every 6 months to do a blood test and then they write a note for my GP and I get my prescription

from my own GP….In between I don’t see anyone unless I feel very sick. Or I only go to my local herbalist once a month and get a mixture of herbs to boil and drink as tea….

FACILITATOR NOTE

At the end of the focus group it is important to thank the participants for their contributions and to give them the opportunity to provide anything additional that they feel is relevant and was not part of the discussion already undertaken. Please see proposed wording in blue below:

‘Thank you all for your contribution today-before we finish is there anything relevant you think we haven’t covered or anything else you would like to add?’

Don’t forget to write a short reflective account of how the focus group went, including things like the dynamics of the groups, what went well, what didn’t go so well and why that might have been and anything unexpected that came up.
